# To Our Patrons

**Published:** 1861-06

**Authors:** 


### EDITORIAL AND MISCELLANEOUS.

### To Our Patrons.

We send out with the present issue of the Journal, the accounts for subscription. In these "hard times," we would not dun a single subscriber were it not necessary to their receiving the Journal that we be paid in part the amounts due. We do not intend, however, to suspend the publication if not a dollar is paid for months to come, but if remittances are not made before the next issue, the number of copies will be considerably curtailed. Those who have been in arrears for some time, and seem to manifest no disposition to aid us in the publication, when they are justly indebted the small amounts that might be paid without inconvenience to them, and with so much benefit to us, must not expect the July number, without remitting something. In short, those who will not pay ought not expect us to pay monthly for the publication of the Journal, beside the confinement and labor necessary on our part, and send it regularly, without any remuneration. We ask nothing more than the expenses of publication, and if all of our subscribers would send us one dollar each, and *repeat the dose* as often as they might do without even missing the amount, we could feel an independence which proprietors of periodicals require to insure the necessary spirit and energy for such position. Friends, send on your 5s, 3s, 2s, and even 1s. Send something, do. We cannot willingly strike the name of any one from our list. The very idea of having to do so annoys us exceedingly.

Do try one dollar, if you cannot get your consent to do more, and my word for it, you will feel better, sleep better, and never miss it. Any bank bill will do, if current where you live. Send on before you forget it.

